# Association between Health Insurance Type and Genetic Testing and/or Counseling for Breast and Ovarian Cancer

**DOI:** 10.3390/jpm12081263

**Published:** 2022-07-31

**Authors:** Arian Mansur, Fang Zhang, Christine Y. Lu

**Affiliations:** 1 Harvard Medical School, Boston, MA 02115, USA; arianmansur@hms.harvard.edu; 2 Department of Population Medicine, Harvard Medical School and Harvard Pilgrim Health Care Institute, 401 Park Drive, Suite 401 East, Boston, MA 02215, USA; fang_zhang@harvardpilgrim.org

**Keywords:** genetic testing, genetic counseling, breast and ovarian cancer, health insurance, genomic medicine, precision medicine

## Abstract

As genetic testing becomes increasingly incorporated into clinical practice to aid in both the diagnosis and risk assessment of genetic diseases, patients benefit from genetic counseling to support their understanding of test results either before and/or after genetic testing. Therefore, access to genetic testing and counseling is imperative for patient care. It is well established that health insurance coverage is a major determinant of access to health care in the United States as individuals without insurance are less likely to have a regular source of health care than their insured counterparts. Different health insurance plans and benefits also influence patients’ access to health care. Data on the association of health insurance and the uptake of genetic testing and/or counseling for cancer risk are limited. Using data from the National Health Interview Survey, we examined the uptake of genetic testing and/or counseling for breast/ovarian cancer risk by health insurance type. We found that only a small proportion of women undergo genetic testing and/or counseling for breast/ovarian cancer risk (2.3%), even among subgroups of women at risk due to family or personal history (6.5%). Women with health insurance were more likely to undergo genetic testing and/or counseling for breast/ovarian cancer risk, particularly those with military and private insurance plans, than those without health insurance after adjusting for various demographic, socioeconomic, and health risk covariates. Further investigations are needed to examine potential disparities in access and health inequities.

## 1. Introduction

Since the completion of the Human Genome Project, genomics has transformed the approach to the diagnosis, management, and prevention of disease [1]. Precision medicine, which is often described as providing “the right patient with the right drug at the right dose at the right time,” has greatly benefitted from the advancements in genetic testing and genetic counseling to tailor treatment and guide preventive measures [2,3,4]. For instance, women with a *BRCA1*/2 pathogenic mutation are at a much greater risk of developing breast and ovarian cancer than those in the general population [5,6]. Breast cancer is currently the most common cancer afflicting women worldwide, accounting for roughly one-quarter of all new cancer cases diagnosed in 2020 [7]. Ovarian cancer is less common, but hereditary breast and ovarian cancer (HBOC) syndrome accounts for approximately 90% of the hereditary neoplasms and is highly associated with *BRCA1/2* mutations [8,9]. The early identification and subsequent interventions (e.g., mastectomy or oophorectomy) can significantly decrease risk and improve mortality and morbidity [10,11]. However, in order to effectively utilize and continue to improve precision medicine requires the recognition of potential sources of underperformance. 

While genetic testing and counseling may help assess individuals’ cancer risk and guide appropriate next steps, it is well known that these genetic services remain under-utilized in the United States [12,13,14]. In fact, a relatively recent study looked at all women at least 20 years of age diagnosed with breast or ovarian cancer in California and Georgia, two large and diverse states, between 2013 and 2014 that were reported to the surveillance, epidemiology, and end results registries and demonstrated that less than one-quarter of women diagnosed with breast cancer and less than one-third of women diagnosed with ovarian cancer underwent any genetic testing despite 8–15% of them having actionable pathogenic variants [12]. They also found variations in testing by health insurance, race/ethnicity, and marital status [12]. Therefore, despite having many guidelines and well-defined strategies for screening, genetic services continue to remain underutilized. 

Genetic testing can assess individuals’ cancer risk and guide screening and preventive measures, but access to genetic services is likely influenced by individuals’ health insurance in the United States. The United States health system is a mix of public and private, for-profit and nonprofit insurers, with about 8.5% of the population uninsured in 2020 [15]. The national Medicare program covers care for adults aged 65 and older and some people with disabilities. There are also various federal programs for veterans and low-income people, including Medicaid and the Children’s Health Insurance Program, with the states managing and paying for local coverage. Private insurance is the dominant form of insurance for nonelderly adults and is provided primarily by employers. However, there are wide variations in insurance coverage of genetic services. While most plans cover genetic testing that is recommended by a physician, the exact policies on coverage and reimbursements depend on the type of health insurance, and costs vary between several hundred to thousands of USDs per test [16,17]. Coverage policies are not available for all forms of genetic testing, and the existing ones are inconsistent across insurers, with a lack of transparency [18]. Concerns about genetic discrimination may also influence the utilization of genetic testing and related services, as individuals often worry that their genetic results will be adversely used to deny coverage or determine premiums, despite the Genetic Information Non-discrimination Act (GINA) that prohibits such discrimination by employers and health insurance companies [19].

It is well known that health system (e.g., health insurance) levels, specific insurance coverage policies, and the magnitude of patient cost-sharing affects access to genomic testing and counseling [20,21,22,23,24,25]. However, there is a lack of data specific to breast and ovarian cancer. The objective of this study was to examine the association between the uptake of genetic testing and/or genetic counseling for breast and/or ovarian cancer risk and the type of health insurance while adjusting for potential confounders.

## 2. Materials and Methods

### 2.1. Data Source

Data were extracted from the publicly available Integrated Public Use Microdata Series National Health Interview Survey (NHIS) project that obtains census data from the NHIS [26]. The NHIS is an annual survey conducted by the U.S. Census Bureau on behalf of the National Center of Health Statistics, which is part of the U.S. Centers for Disease Control and Prevention. The NHIS constitutes the primary source of data on the health status and health care access of the civilian noninstitutional population and has been influential in tracking progress towards the health objectives of the nation [27]. Given that this study included publicly available data and did not involve “human subjects,” an institutional review board review or ethical clearance was not required.

Our analysis was limited to 2015 since that was the latest Cancer Control Supplement, which contains data on individual genetic testing behavior, available with breast and ovarian cancer data at the time of the study. 

### 2.2. Data Measures

Our main outcome variable assessed genetic testing and/or genetic counseling for breast and/or ovarian cancer risk. Our control group were those who answered “No” to both survey questions: “*Have you EVER HAD a genetic test to determine if you are at greater risk of developing cancer in the FUTURE? This does not include any test to see whether you had cancer in the PAST or have cancer NOW.*” and “*These next questions refer to genetic COUNSELING for cancer risk. We will ask about genetic TESTING for cancer risk in a few minutes. Genetic counseling involves a discussion with a specially trained health care provider about your family history of cancer and how likely you are to develop cancer. It may also include a discussion about whether genetic testing is right for you. Have you ever received genetic counseling for cancer risk?*”. Those with genetic testing and/or genetic counseling for breast and/or ovarian cancer risk were those who answered “Yes” to the above-mentioned survey questions, in addition to answering “Yes” to either question specific to breast cancer and/or ovarian cancer: “*Please think about your MOST RECENT genetic test for cancer risk. Was it for breast cancer?*”, “*Please think about your MOST RECENT genetic test for cancer risk. Was it for ovarian cancer?*”, “*Please think about your MOST RECENT genetic counseling session for cancer risk. Was it for breast cancer?*” and “*Please think about your MOST RECENT genetic counseling session for cancer risk. Was it for ovarian cancer?*”.

Our primary predictor of interest was health insurance type. Similar to a previous study [28], health insurance types included ‘no insurance,’ ‘Medicaid,’ ‘Medicare,’ ‘Military,’ ‘Dual’ [Medicaid and Medicare], ‘Other Public,’ and ‘Private’. ‘Military’ includes health insurance coverage through some form of military health insurance, e.g., Veteran Affairs (VA) health insurance, and ‘Other Public’ includes health care coverage provided by a public program other than ‘Medicare,’ ‘Medicaid,’ and ‘Military’ (e.g., state-sponsored health insurance). In our preliminary analysis, there were no meaningful differences between ‘Private’ health insurance with or without a high deductible (possibly due to the sample size), and, therefore, we created only one group of individuals with any ‘Private’ insurance. Individuals with unknown health insurance were excluded from the analysis. 

Demographic characteristics included age, sex, race, marital status, education level, and income level. Other predictors of interest included a personal history of breast and/or ovarian cancer, family history of breast and/or ovarian cancer, self-perceived risk of breast cancer (self-perceived risk of ovarian cancer was not available), and chronic conditions. A self-perceived risk was assessed by participants’ responses to the question, “Compared to the average woman your age, would you say that you are more likely to get breast cancer, less likely, or about as likely? For a breast cancer survivor, this means getting breast cancer again in the future.” Response options included “More likely,” “Less likely,” “About as likely,” or “Don’t know.” ‘Chronic conditions’ was defined as having had at least one of the various following conditions: hypertension, coronary heart disease, diabetes, cancer (not including breast or ovarian cancer), stroke, chronic bronchitis, emphysema, current asthma, and kidney disease [29]. 

### 2.3. Data Analysis

Given the complex survey design of the NHIS, statistical analyses were adjusted with sampling weights and variance estimation methodologies using the survey module in StataMP, version 17.0 for Mac (StataCorp, College Station, TX, USA). Descriptive statistics were created for weighted samples. Comparisons between those with versus those without genetic testing and/or counseling were evaluated using the Student’s *t*-test or Wilcoxon rank sum test for continuous variables and the Pearson’s χ^2^ test or Fisher’s exact test for categorical variables. Simple and multivariable logistic regression models were generated to examine the association between genetic testing and/or counseling for breast/ovarian cancer risk and health insurance type. This analysis was repeated in a subgroup of ‘at-risk’ women with either a personal history of breast and/or ovarian cancer, family history of breast and/or ovarian cancer, or believed that their risk of breast and/or ovarian cancer was more likely when compared to an average person of the same age (*n* = 5122). 

## 3. Results

**Figure 1:**
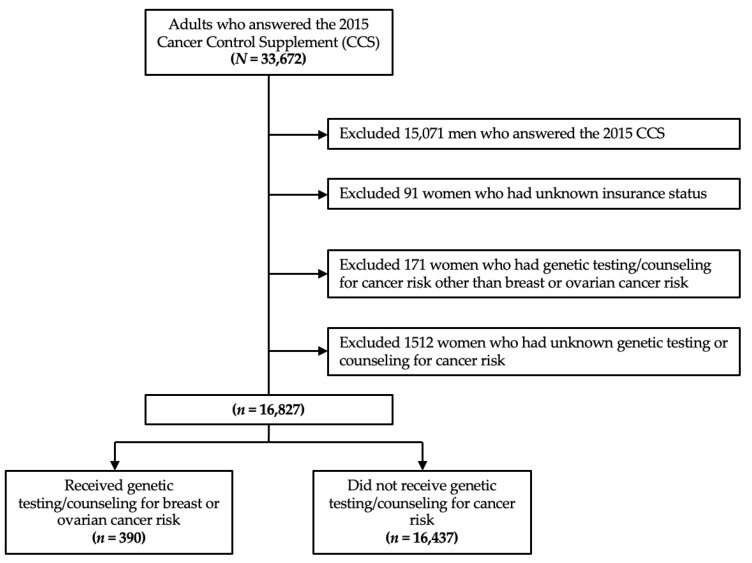
Study flow diagram.

Of 16,827 women that met the study criteria, 390 people (2.3% weighted) received genetic testing and/or counseling for breast/ovarian cancer risk (Figure 1). Among 5122 at-risk women, 337 women (6.5% weighted) received genetic testing and/or counseling for breast/ovarian cancer risk. Among 667 at-risk women who had a personal history of breast/ovarian cancer, 140 (20.6% weighted) obtained genetic testing and/or counseling for breast/ovarian cancer risk. Table 1 summarizes the baseline characteristics of our study cohort. Those who received genetic testing/counseling for breast/ovarian cancer risk were older, more likely to be married or living with a partner, have gone to college, more likely to be above the poverty line, have a personal and family history of breast/ovarian cancer, viewed themselves as more likely to get breast cancer compared to an average woman of the same age, were more likely to have at least one chronic condition, and were more likely to have some form of health insurance (Table 1). 

Table 2 shows the results from the univariable and multivariable logistic regression models estimating the association between genetic testing and/or counseling for breast/ovarian cancer risk and health insurance type. In the unadjusted analysis, women with ‘Medicare,’ ‘Military,’ ‘Dual,’ and ‘Private’ health insurance were significantly more likely to have had an uptake of genetic counseling and/or testing for breast/ovarian cancer risk than those without health insurance. In the adjusted analysis, women with ‘Military’ and ‘Private’ health insurance plans were significantly more likely to have had genetic counseling and/or testing for breast/ovarian cancer risk than those without any health insurance.

Among the subgroup of women who had either a personal history of breast/ovarian cancer, family history of breast/ovarian cancer, or believed that their risk of breast cancer was more likely than an average woman of the same age, the unadjusted analysis found that women with ‘Medicare,’ ‘Military,’ ‘Dual,’ and ‘Private’ health insurance types were significantly more likely to have had an uptake of genetic counseling and/or testing for breast/ovarian cancer risk than those without health insurance (Table 3). In the adjusted analysis, women with ‘Military,’ ‘Dual,’ and ‘Private’ health insurance plans were significantly more likely to have had genetic counseling and/or testing for breast/ovarian cancer risk than those without any health insurance (Table 3).

## 4. Discussion

In this national analysis of adult self-reported survey data, we found that a small proportion of women undergo genetic testing and/or counseling for breast/ovarian cancer risk, even among subgroups of women at risk due to family or personal history (6.5%). However, a higher proportion (20.6%) of women with a personal history of breast/ovarian cancer underwent genetic testing and/or counseling for breast/ovarian cancer risk (temporal relationships cannot be made due to the survey nature of NHIS). These low rates of genetic testing and genetic counseling at a population level are similar to previously reported studies using NHIS survey data [14,30]. While only an estimated 10% of breast and ovarian cancers result from hereditary causes, which may explain our finding of a low rate of genetic testing and counseling for breast/ovarian cancer risk, it has been extensively shown that these genetic services are underutilized and current testing guidelines miss clinically actionable identification [12,31,32,33].

The United States Preventative Services Task Force currently recommends genetic counseling, and if indicated after counseling, genetic testing only for women with a personal or family history of breast, ovarian, tubal, or peritoneal cancer or an ancestry associated with the *BRCA1/2* gene mutation [34]. Similarly, the National Comprehensive Cancer Network recommends these genetic services based on a personal or family history and how closely related a person is to the person(s) who developed cancer or those who have inherited cancer predisposition disorders, such as Li-Fraumeni syndrome [35]. The American Society of Clinical Oncology recommends that all women with epithelial ovarian cancer should obtain genetic testing for inherited variants in *BRCA1/2* and similar susceptible genes without regard to their family history [36]. Our study and others suggest that increased efforts are still needed to improve the uptake of genetic testing and genetic counseling for improving the treatment, management, and prevention of breast and ovarian cancer, as well as public health.

We identified an association between genetic testing and/or counseling for breast/ovarian cancer risk and type of health insurance, adjusting for various demographic, socioeconomic, and health risk covariates. We found that women with ‘Military’ and ‘Private’ health insurance plans were significantly more likely to receive genetic testing and/or counseling for breast/ovarian cancer risk than those with no health insurance. Importantly, in a subgroup analysis limited to at-risk women for breast/ovarian cancer, we also found that the uptake of genetic testing and/or counseling differed by health insurance type. In addition to ‘Military’ and ‘Private’ health insurance, women with ‘Dual’ [Medicare and Medicaid] were significantly more likely to have had genetic testing and/or counseling when compared to those with no health insurance. The statistically insignificant results for some of the health insurance types in the multivariable regression models were most likely due to the lower rates of uptake after adjusting for covariates. While it is encouraging that some women with health insurance, particularly those at-risk of breast/ovarian cancer and who would benefit from its access, have greater access to genetic testing/counseling for breast/ovarian cancer risk than those uninsured, our findings suggest that access likely differed across insurance types. It is well known that there are complexities in the United States health insurance coverage plans, as well as variations in access to health services [37,38,39,40]. These complexities and the variation in access further extend to include genetic services, especially as health insurance coverage policies do not exist for all types of genetic testing. Existing coverage policies differ substantially across insurers for guideline-recommended pharmacogenetic tests and genetic tests that identify hereditary cancer risk [26,41]. Increasing evidence encourages the utilization of germline cancer tests in patients who are evaluated for hereditary cancer [42,43], as these results can better inform cancer screening recommendations and further surgical considerations. The evidence suggests that health insurance coverage policies may not meaningfully differentiate between patients with cancer who are likely to benefit from germline genetic testing for cancer risk and those who are unlikely to [18].

There have been other studies that used the NHIS to study the uptake of genetic services for breast and/or ovarian cancer [14,30]. Allen et al. examined the associations of familial cancer risk for different genetic services, including genetic testing/counseling, but they found no association between the uptake of genetic testing or genetic counseling and health insurance status [14]. This finding is counterintuitive and conflicts with ours. It is worth noting, however, that they examined genetic testing/counseling for all cancer types while stratifying by the level of familial risk, and their health insurance variable was limited to “Private” versus “Other,” as the insurance was not the focus of their work. Our findings also conflict with those of Turbitt et al., who found no association between health insurance and the uptake of genetic services or genetic counseling for breast cancer [30]. However, they excluded individuals with a personal cancer history, did not examine ovarian cancer, and they did not use an expanded version of the insurance type variable and, instead, used a binary (covered versus not covered, as the insurance was not the focus of their work) [30]. The heterogeneity in these studies, especially their methods, makes comparisons difficult.

There are several strengths to our study. It is one of the first to systematically analyze the various types of health insurance and their association with the uptake of genetic services for breast/ovarian cancer risk. Furthermore, by utilizing the NHIS, we were able to generate a larger cohort that was representative of the national population and limited confounders. However, there are still several limitations to the present analysis. As a cross-sectional analysis, we cannot make statements about the temporal relationships between the insurance types and uptake of genetic testing and/or counseling for breast/ovarian cancer risk. Since our study was observational in nature, we also cannot make any causal claims, and there is still the possibility of confounding variables that were not accounted for. We attempted to minimize bias by performing a subgroup analysis limited to women who were at-risk for breast/ovarian cancer (a more homogenous group) and for whom health insurance policies are likely to be more similar. While the NHIS does collect some information about patient cost-sharing, we were unable to examine its interactive effects with health insurance due to the sample size. We are also unable to examine the separate associations of the health insurance type with the specific genetic services due to insufficient statistical power, and we created a composite outcome variable that combined genetic testing and/or counseling for breast and/or ovarian cancer risk. Furthermore, our variables were limited by the design of NHIS. For example, we were not able to identify women who have family members with inherited cancer syndromes (who might also benefit from genetic testing and/or counseling based on clinical guidelines), which is why we included a family history of breast/ovarian cancer. Finally, the self-reported survey data may be susceptible to recall bias [44].

## 5. Conclusions

The findings from this inferential analysis suggest variations in the access to genetic services for breast/ovarian cancer risk across different health insurance types. The current lack of standardization in health insurance coverage policies for genetic services may present a significant barrier for women who could otherwise benefit from such services. Further investigations are needed to examine the potential disparities in access and health inequities. Increasing efforts to align health insurance coverage policies with clinical guidelines, and to minimize variations in insurance coverage policies across insurers for genetic testing and/or counseling, are needed. These efforts will allow for the equitable integration of genetic services in the clinical setting.

## Figures and Tables

**Table 1:** Baseline characteristics.

Variable	Received Genetic Testing/Counseling	Did Not Receive Genetic Testing/Counseling	p *
Unweighted *N* (weighted %)	390 (2.3)	16,437 (97.7)	
Insurance Type, unweighted No. (weighted %)			<0.01
Uninsured	17 (3.7)	1548 (8.3)	
Medicaid	35 (6.2)	1948 (10.5)	
Medicare	87 (23.3)	3850 (23.7)	
Military	24 (6.2)	555 (3.5)	
Dual	21 (4.6)	672 (3.6)	
Other Public	2 (0.3)	164 (1.0)	
Private	204 (55.9)	7700 (49.6)	
Age (years), weighted mean (SE)	52.6 (0.99)	50.3 (0.24)	<0.01
Race, unweighted No. (weighted %)			0.12
White	307 (82.1)	12,514 (79.0)	
Black	57 (13.3)	2426 (13.4)	
Other	26 (4.7)	1497 (7.6)	
Marital Status, unweighted No. (weighted %)			0.01
Married/live with partner	205 (55.7)	7756 (47.3)	
Not currently married^1^	184 (44.3)	8645 (52.4)	
Unknown	1 (0.1)	36 (0.3)	
Education, unweighted No. (weighted %)			<0.01
Less than college	100 (26.8)	6257 (35.6)	
College	286 (72.4)	10,120 (64.0)	
Unknown	4 (0.9)	60 (0.4)	
Combined Family income, Unweighted No. (weighted %)			<0.01
At or above poverty line	334 (87.3)	12,807 (79.6)	
Below poverty line	47 (10.1)	2845 (15.7)	
Unknown	9 (2.5)	785 (4.7)	
Personal History of BOC, unweighted No. (weighted %)			<0.01
Yes	140 (36.5)	527 (3.3)	
No	250 (63.5)	15,910 (96.7)	
Family History of BOC, unweighted No. (weighted %)			<0.01
Yes	267 (69.7)	3911 (23.8)	
No	123 (30.4)	12,526 (76.2)	
Self-perceived BC risk, unweighted No. (weighted %)			<0.01
Less likely	85 (24.8)	6459 (38.5)	
About as likely	116 (29.4)	7398 (45.7)	
More likely	178 (43.2)	1722 (10.6)	
Unknown	11 (2.6)	858 (5.2)	
Chronic conditions, unweighted No. (weighted %)			<0.01
None	117 (28.7)	8105 (50.2)	
At least 1	273 (71.3)	8300 (49.6)	
Unknown	0 (0)	32 (0.2)	

Abbreviations: BOC, breast and/or ovarian cancer; No., number; SE, standard error. ^1^ Included people who are widowed, divorced, separated, or never married. * *p*-values were adjusted for sampling weights.

**Table 2:** Logistic regression evaluating association between the uptake of genetic testing/counseling for breast and/or ovarian cancer and health insurance type.

Variable	OR (95% CI)	*p*	aOR (95% CI)	*p*
Insurance type (ref = uninsured)				
Medicaid	1.33 (0.63–2.84)	0.46	0.99 (0.43–2.28)	0.98
Medicare	**2.23 (1.22–4.07)**	**<0.01**	1.08 (0.51–2.26)	0.82
Military	**4.07 (1.90–8.71)**	**<0.01**	**3.45 (1.49–8.00)**	**<0.01**
Dual	**2.96 (1.38–6.38)**	**<0.01**	1.65 (0.69–3.91)	0.26
Other Public	0.66 (0.13–3.23)	0.60	0.72 (0.14–3.85)	0.70
Private	**2.55 (1.39–4.70)**	**<0.01**	**2.16 (1.11–4.20)**	**0.02**
Age (per year)	–	–	0.99 (0.98–1.00)	0.15
Race (ref = white)				
Black	–	–	1.42 (0.96–2.12)	0.08
Other	–	–	0.70 (0.40–1.23)	0.22
Married/live with partner versus not currently married ^1^	–	–	0.74 (0.54–1.01)	0.06
Education less than college versus college	–	–	1.19 (0.85–1.66)	0.31
Household Income at or above versus below poverty line	–	–	1.06 (0.64–1.75)	0.83
No versus at least one chronic condition			1.10 (0.78–1.54)	0.59
Personal history of BOC versus no history	–	–	17.2 (11.5–25.6)	<0.01
Family history of BOC versus no history	–	–	6.40 (4.76–8.59)	<0.01
Perceived breast cancer risk in self (ref = less likely)				
About as likely	–	–	0.75 (0.51–1.10)	0.14
More likely	–	–	1.68 (1.15–2.46)	<0.01

Abbreviations: BOC, breast and ovarian cancer; No., number; SE, standard error. ^1^ Included people who are widowed, divorced, separated, or never married. Bold indicates statistical significance (*p* < 0.05) in the insurance type variable.

**Table 3:** Logistic regression evaluating association between the uptake of genetic testing/counseling for breast and/or ovarian cancer and health insurance type for at-risk women.

Variable	OR (95% CI)	*p*	aOR (95% CI)	*p*
Insurance type (ref = uninsured)				
Medicaid	1.82 (0.68–4.83)	0.23	1.92 (0.73–5.06)	0.19
Medicare	**2.75 (1.19–6.35)**	**0.02**	2.25 (0.95–5.32)	0.07
Military	**6.06 (2.29–16.09)**	**<0.01**	**5.00 (1.86–13.48)**	**<0.01**
Dual	**3.70 (1.43–9.59)**	**<0.01**	**3.81 (1.50–9.72)**	**<0.01**
Other Public	0.58 (0.07–5.02)	0.62	0.62 (0.07–5.42)	0.67
Private	**4.09 (1.75–9.53)**	**<0.01**	**3.19 (1.35–7.54)**	**<0.01**
Age (per year)	–	–	1.00 (0.99–1.02)	0.36
Race (ref = white)				
Black	–	–	1.39 (0.96–2.03)	0.08
Other	–	–	0.76 (0.42–1.38)	0.37
Married/live with partner versus not currently married ^1^	–	–	0.71 (0.52–0.97)	0.03
Education less than college versus college	–	–	1.26 (0.90–1.76)	0.18
Household Income at or above versus below poverty line	–	–	0.73 (0.43–1.22)	0.23
No versus at least one chronic condition			0.84 (0.67–1.33)	0.74

Abbreviations: No., number; SE, standard error. ^1^ Included people who are widowed, divorced, separated, or never married. Bold indicates statistical significance (*p* < 0.05) in the insurance type variable.

## Data Availability

The data presented in this study are publicly available and can be downloaded from https://www.cdc.gov/nchs/nhis/nhis_2015_data_release.htm (accessed on 15 November 2021).
